# Gastrokine 1 transferred by gastric cancer exosomes inhibits growth and invasion of gastric cancer cells in vitro and in vivo

**DOI:** 10.1002/ccs3.12044

**Published:** 2024-07-07

**Authors:** Lingling Tian, Li Tang, Xu Li, Liuye Huang

**Affiliations:** ^1^ Department of Gastroenterology Yantai Yuhuangding Hospital Shandong University Yantai Shandong China

**Keywords:** exosomes, gastric cancer, Gastrokine 1, invasion, PI3K/AKT/mTOR

## Abstract

In gastric cancer, gastrokine 1 (GKN1) is a potential theragnostic marker while the related mechanisms remain elusive. Exosomes mediate intercellular communications via transferring various molecules, yet there are limited research studies on the specific cargos of gastric cancer exosomes and the associated mechanisms in this disease. In the present study, AGS and N87‐C cells were transfected with an overexpressed GKN1 plasmid, followed by extraction of exosomes. The study utilized gastric cancer cell lines and a xenograft mouse model to investigate the functional significance of exosomal GKN1. Cell proliferation, metastasis, and apoptosis were assessed through CCK‐8, Transwell, and flow cytometry assays, respectively. The study further explored the mechanism of exosomal GKN1 and its interaction with the PI3K/AKT/mTOR signaling pathways, including immunofluorescence and western blot analyses. Exosomal GKN1 was observed to suppress cell proliferation and invasion while enhancing apoptosis. This effect was attributed to the modulation of key proteins involved in cellular processes, including Ki‐67, MMP‐9, Bcl‐2, Bax, caspase‐3, and caspase‐9, ultimately impacting the PI3K/AKT/mTOR signaling pathway. The findings suggest that exosomal GKN1 exerts inhibitory effects on gastric cancer cell growth and invasion through the regulation of the PI3K/AKT/mTOR signaling cascade, both in experimental cell cultures and animal models.

## Graphical abstract

Gastrokine 1 transferred by gastric cancer exosomes inhibits growth and invasion of gastric cancer cells both in vitro and in vivo via regulating PI3K/AKT/mTOR signaling pathway.

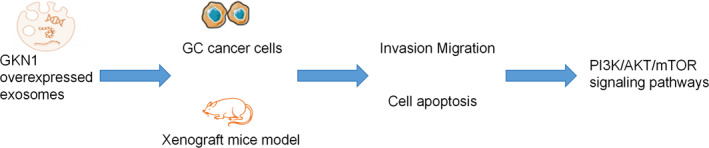

## 1 INTRODUCTION

Gastric cancer ranks as the fifth most deadly malignant tumor of the digestive system on a global scale.^1^, ^2^ The elevated occurrence of gastric cancer is predominantly attributed to chronic *helicobacter pylori* infection, lifestyle stress, and inadequate dietary habits. Presently, the primary therapeutic approach encompasses surgical intervention, postoperative chemotherapy, and biological immunotherapy.^3^, ^4^ Due to the challenges associated with early diagnosis of gastric cancer, including aggressive local invasion and early metastasis, it continues to pose a significant threat to human health.^5^, ^6^ Therefore, finding targeted factors and discovering new diagnostic biomarkers are essential.

An exosome is a small nanovesicle, composed of lipid bilayer and ranges in size between 30 and 100 nm.^7^ Recent research studies have shown that exosomes contain functional biomolecules, including cell‐derived proteins, lipids, glycoconjugates and nucleic acids.^8^, ^9^ Exosomes can be transported between recipient cells, and therefore regulate the cell microenvironment and immune system. With the study of multiple roles of exosomes, the potential functions of exosomes in regulating the tumor microenvironment have been reported.^7^, ^10^ Moreover, exosomes participated in tumorigenesis promotion, metastasis, angiogenesis, and tumor therapy resistance.^11^ Exosomes derived from tumor cells have been reported to regulate the microenvironment and immune system in recent years.^12^, ^13^ As shown in the recent studies, human bone marrow mesenchymal stem cells can be activated by glioma cell‐derived exosomes to regulate tumor‐like phenotype transformation. Additionally, exosomes derived from gastric cancer cells could affect tumor growth and angiogenesis in the NOD/SCID mouse, promoted gastric cancer cell proliferation via regulating PI3K/Akt and MAPK/ERK pathways.^14^, ^15^

Gastrokine‐1 (GKN1), a stomach‐specific protein containing 185 amino acid residues, was produced by gastric mucosal epithelium, epithelial metaplasia tissues, and smooth muscle tissues; meanwhile, exosomal cargo proteins may be released into the extracellular environment.^16^, ^17^ The increased levels of GKN1 in gastric cells could protect the mucosa from injury, thereby promoting the repair of injured mucosa and inhibiting tumor growth.^18^ According to other studies, GKN1 is highly expressed in normal mucosal tissues but is lost in gastric cancer cells, suggesting that that GKN1 expression may be a diagnostic marker for gastric cancer.^19^, ^20^

The exosomal GKN1 protein isolated from immortalized gastric epithelial cells has been shown to regulate cell viability and apoptosis both in vivo and in vivo. Additionally, exosomes containing GKN1 reduce tumor volume and weight and inhibit gastric cancer by downregulating HRas/Raf/MEK/ERK signals.^21^ A significant reduction in tumor volume and tumor weight was observed in exosomes containing GKN1‐treated nude mice.^22^ GKN1 derived from gastric tumor cells, however, did not appear to be used in gastric cancer treatment.

Based on the aforementioned literature, in the present study, AGS and N87‐C cells were transfected with an overexpressed GKN1 plasmid, followed by extraction of exosomes. This study investigated the effects of gastric cancer‐derived exosomal GKN1 on AGS and N87‐C cells, as well as a mouse model. Furthermore, the study explored the impact of GKN1 on gastric cancer xenograft tumor mice, specifically focusing on inflammation, cell proliferation, and PI3K/Akt/mTOR signaling pathways. The primary objective of this research study was to identify a potential therapeutic target for further investigation of exosomal GKN1 in gastric cancer.

## 2 MATERIALS AND METHODS

### 2.1 Cell culture and transfection

We purchased human gastric cancer cell lines AGS and N87‐C from BeNa Culture Collection (Henan, China, BNCC341304). The cell lines were maintained in Dulbecco's modified Eagle's medium (DMEM) medium containing 10% fetal bovine serum (FBS). Penicillin G and streptomycin sulfate were added at 100 U/mL to the medium to avoid bacterial resistance. At 37°C and 5% CO_2_, all cells were cultured in the humid incubator.

We obtained the overexpressed GKN1 plasmid and its control From Hanbio Biotechnology (Shanghai, China). The Lipofectamine 2000 transfection reagent (Invitrogen, Calsbad, CA) was used to transfect AGS and N87‐C cells with GKN1 mimic and its negative control. A western blot assay was performed 2 days after transfection to determine GKN1's transfector efficiency.

### 2.2 Isolation and identification of exosomes

The protocol of exosome isolation was conducted by ultracentrifuge based on the previous studies. To remove residual cells and debris, the AGS and N87‐C cells were harvested after 3 days (30 mL) and centrifuged for 10 min at 300 g, 2 min at 2000 g, and 30 min at 10, 000 g, respectively. Following ultracentrifugation at 100,000 g for 70 min, the pellet was resuspended in 50–100 mL PBS.

A Nanosight LM10 System (Nanosight Ltd) equipped with a fast video capture was used to measure exosome size. To calculate nanoparticle concentrations and size distributions, particle tracking software measures Brownian motion. Also, an image of exosomes was obtained by transferring glutaraldehyde to a carbon‐coated copper grid, staining it with 2% uranylactate, drying it, and then imaging it with a transmission electron microscope. To detect exosome surface markers, we used Western blotting. All steps were performed as directed by the manufacturer.^23^ Detection of exosome uptake in AGS and N87‐C cells was conducted using the Nikon Eclipse Ti confocal laser scanning microscope (Nikon).

### 2.3 Cell viability assay

In order to assess the viability of AGS and N87‐C cells, Cell Counting Kit 8 (CCK‐8, Thermo) was employed. Briefly, the AGS and N87‐C cells were seeded into 96‐well plates at a density of 5.0 × 10^3^ cells per well. After adding 10 μL of CCK‐8 solution, absorbance values were measured at 24, 48, and 72 h after adding the solution.

### 2.4 Cell invasion assay

Transwell invasion analysis was performed to detect the AGS and N87‐C cell invasionrate. The transwell chambers (Boyden Chambers, Corning) were used in accordance with the manufacturer's instructions. Accordingly, AGS and N87‐C cells were inducted with the exosomes or other factors and then resuspended in a serum‐free medium containing bovine serum albumin (BSA).

Each upper chamber contained 5 × 10^5^ cells/mL (the inner bottom was coated with matrigel matrix). Growth medium containing 10% serum was filled into the lower chamber in amounts of 500 μL. Formaldehyde was sprayed on the cells of the upper chamber and cotton swabs were used to wipe them clean. An inverted microscope was used to measure the invasiveness of cells on the below side after staining them with 0.5% crystal violet stain.^24^

### 2.5 Cell apoptosis assay

For apoptosis detection, apoptosis detection kits containing Annexin V‐fluorescein isothiocyanate (FITC) as well as propidium iodide (PI) were employed in different experimental groups. AGS and N87‐C cells in the logarithmic growth phase were collected by trypsin and rinsed with PBS before inoculation into a six‐well plate. Annexin V‐FITC and PI added to the sample for 15 min in the dark, and flow cytometry was then used to detect the samples. In accordance with the manufacturer's instructions and previous studies, all procedures were performed.^25^

### 2.6 Immunofluorescence analysis

Immunofluorescence was used to detect GKN1, Ki67, and MMP‐9 expression. Photographs of GKN1 positive cells were taken using fluorescence microscopy. Placing the cells overnight, treating them with either DMSO or RSL3 for 2 h, fixing them in 4% paraformaldehyde, and permeabilizing them in 0.1% Triton was conducted. Blocking of the slides in 0.5% BSA was performed overnight at 4°C, followed by washing in PBS and incubation with secondary antibodies for 1 hour at room temperature. DAPI was used to mount slides in Vectashield. Cells were trypsinized and plated for 2 hours in 60 mm low adherent plates for ECM detached experiments. A Zeiss or Nikon A‐1 confocal microscope was used to image the slides.^26^

### 2.7 Establishment of xenograft mice model

A Xenograft mouse model was established by injecting 1 × 10^6^ AGS cells into the left axilla of 40 BALB/c nude mice. Then, after the tumor formation, the mice were randomly divided into four groups (*n* = 10): Control group, GKN1 group, GKN1‐NC group and Exosome group. Intravenous injections at 2 mg exosomes per mouse were performed through tail vein, for one dose every other day and lasts for 2 weeks. Tumor volumes and tumor growth rates were calculated using an electronic Vernier caliper. Using a digital caliper, tumor volume was measured as follows: TV (mm^3^) = length × width^2^ × 0.5.

Animal procedures were approved by Yantai Yuhuangding hospital during experimental studies. ARRIVE guidelines (Animal Research: Reporting of In Vivo Experiments) provided guidelines for reporting in vivo experiments.

### 2.8 Terminal deoxynucleotidyl transferase deoxyuridine triphosphate nick end labeling staining assay

Cell apoptosis was assessed using the TUNEL Apoptosis Detection kit (KeyGen Biotech), as described in previous studies.^27^ All procedures were conducted according to instructions provided by the manufacturer and previous research studies. The images were taken using fluorescence microscopy at a magnification of 20 × in order to identify apoptotic cells.

### 2.9 Immunohistochemical analysis

We detected the expression of GKN1 by immunofluorescence using a Deacetylase Fluorometric Assay kit (Bio Vision Inc., Milpitas Blvd) for measuring deacetylase. Observations of apoptotic cells were conducted by fluorescent microscopy at 20 × magnification.

### 2.10 Western blotting

Equal amounts of proteins were separated from lysed cells in RIPA buffer containing proteinase inhibitors, resolved by a 12% SDS‐PAGE gel. Electrophoresis of proteins was followed by transfer to PVDF membranes, blocking with 5% non‐fat milk, and overnight incubation with primary antibodies. The antibodies targeting specific proteins, including anti‐Bax, anti‐ Bcl‐2, anti‐cleaved caspase‐3, anti‐caspase‐3, anti‐p‐PI3K, anti‐PI3K, anti‐p‐Akt, anti‐Akt, anti‐p‐mTOR, anti‐mTOR, and β‐actin (Cell Signaling Technology), were incubated at a dilution of 1:1000 overnight at 4°C. Following three TBST washes, secondary antibodies were incubated with goat anti‐rabbit or mouse anti‐rabbit HRP conjugates. Afterwards, proteins were visualized using an enhanced chemiluminescence system, and protein density was calculated using Image‐Pro Plus6.0.^15^

### 2.11 Statistical analysis

In order to determine statistical significance, one‐way analysis of variance (ANOVA) followed by Tukey's post‐test was performed using GraphPad Prism version 8.2. Means and standard deviations (SD) were used to represent data and statistically significant differences were defined as *p* < 0.05.

## 3 RESULTS

### 3.1 Characterization of exosomes and identification of GKN1 expressions

**FIGURE 1:**
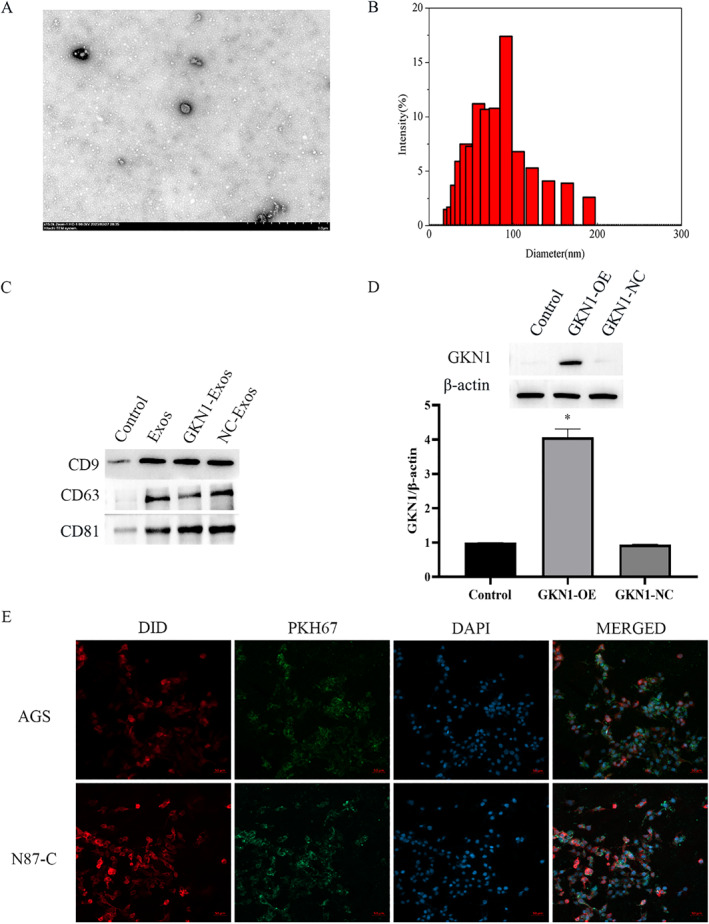
Identification and characterization of exosomes. (A) The morphology of exosomes by TEM; (B) Size distributions of exosomes were measured using nanoparticle tracking analysis (NTA); (C) Expression of exosome‐associated markers (CD9, CD63, and CD81) were detected by Western blot assay; (D) GKN1 expression levels were detected by western blot; (E) Representative fluorescence images of uptake of exosomes by AGS and N87‐C cells; DID (red) and PKH67 (green) staining were used to trace exosomes. *p < 0.05 versus control group.

The characterization of exosomes was based on the results of an electron microscope, NTC, and western blot assays. Based on the results of TEM analysis, showed in Figure 1A, exosomes have cup‐ or sphere‐shaped morphology surrounded by double membranes. Based on DLS measurements, showed in Figure 1B, these particles were mostly 30–100 nm in diameter, as described previously. As seen by western blotting results, showed in Figure 1C, the CD9, CD63, and CD81 expression levels indicated exosomal surface markers. This combination of findings demonstrated that these nanoparticles were exosomes.

Furthermore, the expressions of GKN1 were detected, as shown in Figure 1D. Consistent with the expressions in GC cells, the levels of GKN1 were higher in GKN1‐over expressed group (*p* < 0.05) while the GKN1‐NC group did not show any significant differences from the control group.

Then, in order to detect the effects of GKN1‐overexpressed exosomes on AGS and N87‐C cells, we detected the cell uptake of exosomes. As shown in Figure 1E, the immunofluorescence image certificated that the exosomes uptake was successful both in AGS and N87‐C cells.

### 3.2 GKN1 overexpressed exosomes regulated the cell viability, cell apoptosis and invasion of AGS and N87‐C cells

**FIGURE 2:**
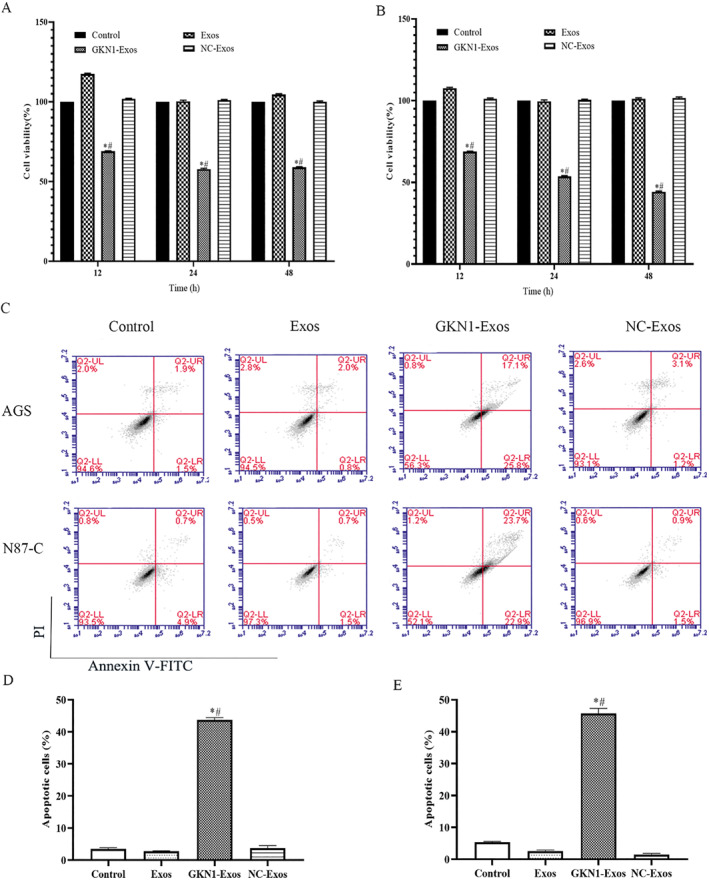
GKN1 overexpression attenuated cell viability and apoptosis rate of AGS and N87‐C cells. (A) The AGS cell viability of each group was detected by CCK‐8 assay; (B) The N87‐C cell viability of each group was detected by CCK‐8 assay; (C) Cell apoptosis rate was detected by flow cytometry assay; (D) AGS cell apoptosis rate; (E) N87‐C cell apoptosis rate; *p < 0.05 versus Control group; #*p* < 0.05 versus Exos group.

**FIGURE 3:**
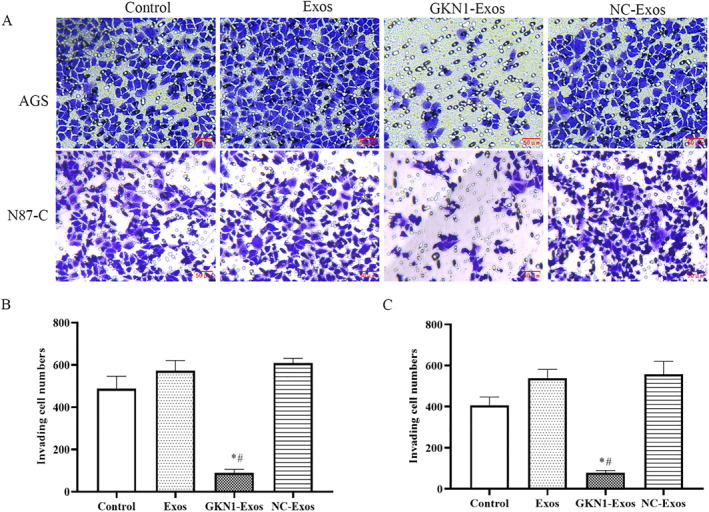
GKN1 overexpression attenuated cell invasion rate of AGS and N87‐C cells. (A) Cell invasion was analyzed with Transwell assay; (B) The AGS cell invasion rate; (C) The N87‐C cell invasion rate; *p < 0.05 versus Control group; #*p* < 0.05 versus Exos group.

To investigate the impact of GKN1 overexpression on cell viability, apoptosis, and invasion rate in AGS and N87‐C cells, these cells were co‐incubated with GKN1 overexpressed exosomes; meanwhile, assays such as CCK‐8, flow cytometry and Transwell assay were performed. As can be seen from Figures 2 and 3, compared to the control group, GKN1‐Exos group showed significantly lower cell viability and invasion rate (*p* < 0.05). Meanwhile, there was no difference between NC‐exo and Exos groups.

Consistent with the cell viability assay results, there was a dramatic increase in apoptosis rates in the GKN1‐ Exos group (*p* < 0.05) while NC‐exo and Exos groups showed no difference.

Taken together, the cell viability, cell apoptosis and invasion rate results revealed that in AGS and N87‐C cells, GKN1‐exos inhibited cell viability, invasion rate, and cell apoptosis.

### 3.3 GKN1 overexpressed exosomes regulated the GKN1 expression in AGS and N87‐C cells

**FIGURE 4:**
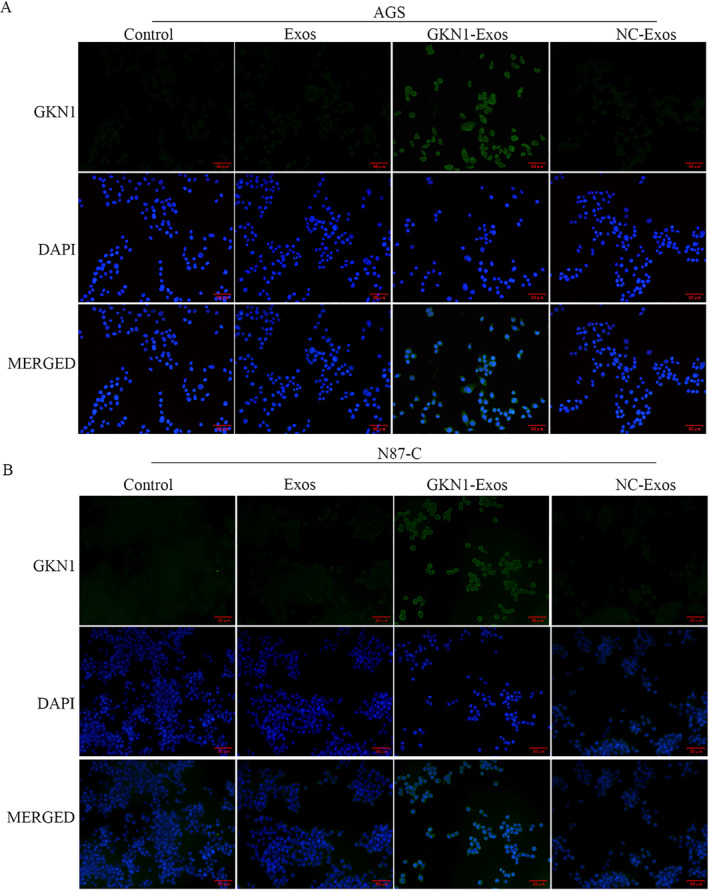
Immunofluorescence staining for GKN1 in AGS and N87‐C cells (scar bar: 20 μm). (A) GKN1 expression in AGS cells, GKN1 (green) and DAPI (blue, nucleus). (B) GKN1 expression in N87‐C cells, GKN1 (green) and DAPI (blue, nucleus).

**FIGURE 5:**
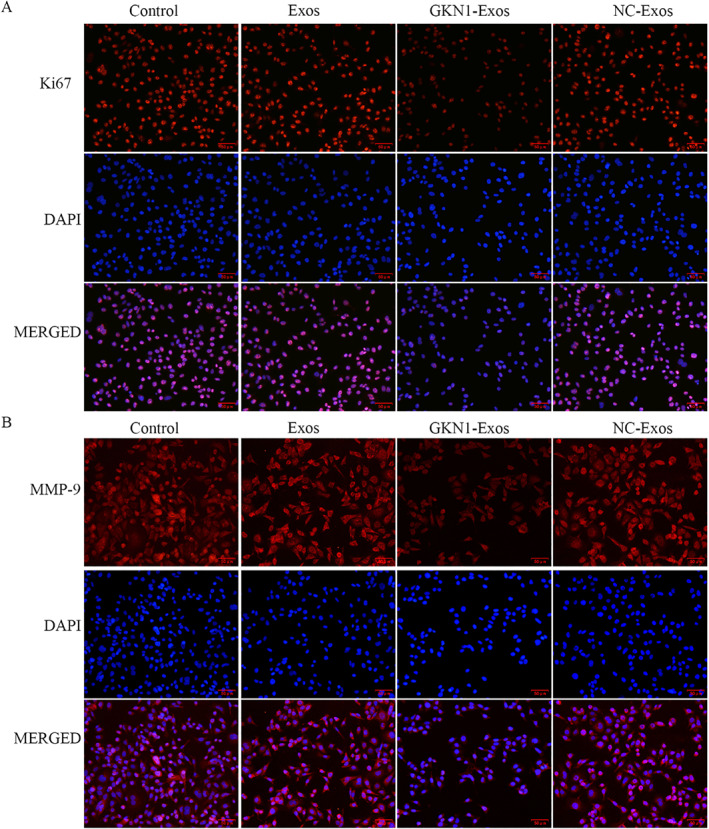
Overexpression of GKN1 attenuated the Ki67and MMP‐9 expressions. (A) Ki67 expression was detected by immunofluorescence assay, Ki67 (red) and DAPI (blue, nucleus). (B) MMP‐9 expressions in was detected by immunofluorescence assay, Ki67 (red) and DAPI (blue, nucleus).

The level of GKN1 expression in AGS and N87‐C cells was assessed using an immunofluorescence assay. The GKN1‐Exos group exhibited higher levels of GKN1 expression compared to the control group, as illustrated in Figures 4 and 5. There was no significant difference in GKN1 expression between the NC‐exo and Exos groups compared to the control group.

### 3.4 GKN1 overexpressed exosomes regulated Ki67 and MMP‐9 expressions in AGS cells

An immunofluorescent staining assay was conducted to assess Ki67 and MMP‐9 expressions in AGS cells. The results indicated that the immunofluorescence intensity of both Ki67 and MMP‐9 expression was significantly higher in the GKN1‐Exos group compared to the control group. No significant difference in immunofluorescence intensity was observed between the NC‐exo and Exos groups compared to the control group, as illustrated in Figure 5.

### 3.5 GKN1 overexpressed exosomes regulated cell apoptosis relative protein levels in AGS cells

**FIGURE 6:**
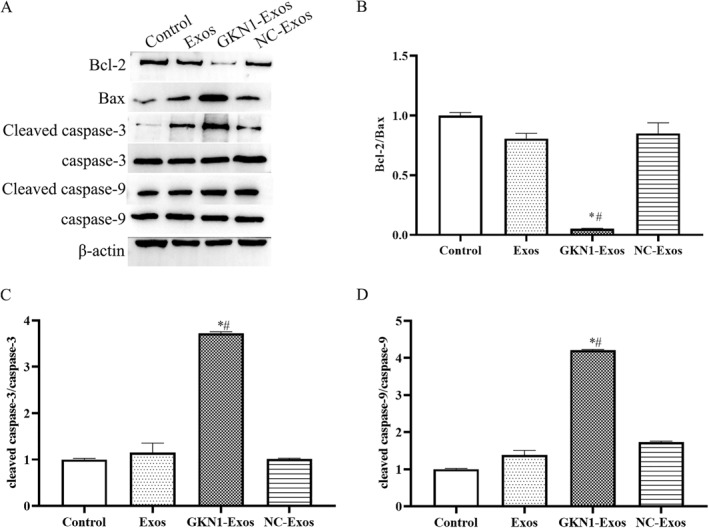
Overexpression of GKN1 attenuated cell apoptosis related indicators expressions. (A) Western blot of cell apoptosis related indicators in tumor cells were performed; (B) Bcl‐2/Bax expression; (C) cleaved‐caspase 3/caspase three expression; (D) cleaved‐caspase 9/caspase nine expression. *p < 0.05 versus Control group; #*p* < 0.05 versus Exos group.

In this study, the levels of cleaved‐caspase‐3/caspase‐3 and cleaved‐caspase‐9/caspase‐9 were measured in AGS cells to investigate the impact of GKN1 on cell apoptosis. Figure 6 illustrates a significant decrease in Bcl‐2/Bax levels in the GKN1‐Exos group compared to the control group (*p* < 0.05), while no notable differences were observed between the NC‐exo and Exos groups compared to the control group. Notably, a significant disparity was observed in the levels of cleaved‐caspase‐3/caspase‐3 and cleaved‐caspase‐9/caspase‐9 between the GKN1‐Exos group and the control group. No notable differences were observed between the NC‐exo and Exos groups compared to the Control group.

The combined effects of GKN1‐Exos resulted in a decrease in Bcl‐2 (antigen of apoptosis) and Bax (pro‐apoptosis), while a significant increase in caspase‐3 cleavage and caspase‐9 cleavage levels were observed. All these results revealed the pro‐apoptotic ability of GKN1‐ Exos in GC cells.

### 3.6 GKN1 overexpressed exosomes inhibited the tumor volume in xenograft mice model

**FIGURE 7:**
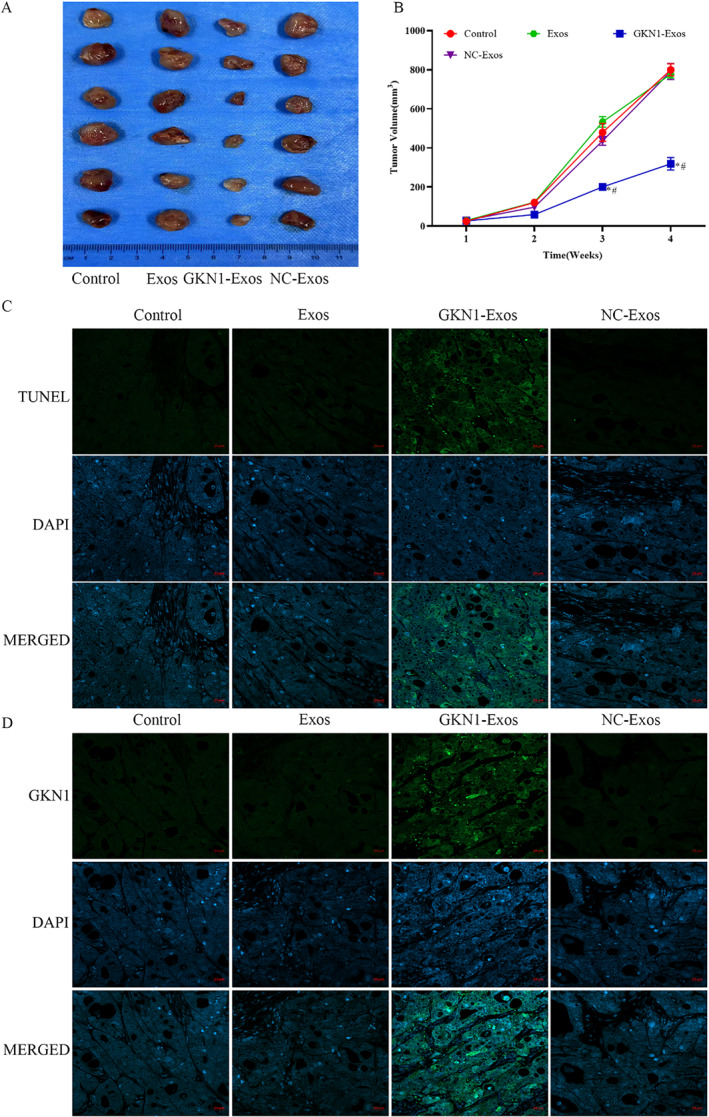
GKN1 overexpression decreased tumor volume and apoptosis in a Xenograft mouse model. (A) Tumor images of AGS cells induced xenograft model; (B) Tumor volume of xenograft model; (C) Tumor cell apoptosis rate was detected by immunofluorescence staining assay, TUNEL (green) and DAPI (blue, nucleus); (D) GKN1 expression was detected by immunofluorescence staining assay, GKN1 (green) and DAPI (blue, nucleus). *p < 0.05 versus Control group; #*p* < 0.05 versus Exos group.

To further assess the impact of GKN1 overexpressed exosomes on tumor growth, an AGS xenograft mice model was established. As depicted in Figure 7A,B, the tumor volume in the GKN1‐Exos group was found to be significantly reduced (*p* < 0.05) compared to the control group, whereas no significant differences were observed in the tumor volumes of the NC‐exo and Exos groups.

### 3.7 GKN1 overexpressed exosomes regulated cell apoptosis rate and GKN1 expression in xenograft mice model

An immunofluorescent staining assay was conducted to assess cell apoptosis and GKN1 expression in tumor tissue. The results indicated that the immunofluorescence intensity of both cell apoptosis and GKN1 expression was significantly higher in the GKN1‐Exos group compared to the control group. No significant difference in immunofluorescence intensity was observed between the NC‐exo and Exos groups compared to the control group, as illustrated in Figure 7C,D.

### 3.8 GKN1 overexpressed exosomes regulated PI3K/Akt/mTOR relative protein levels in xenograft mice model

**FIGURE 8:**
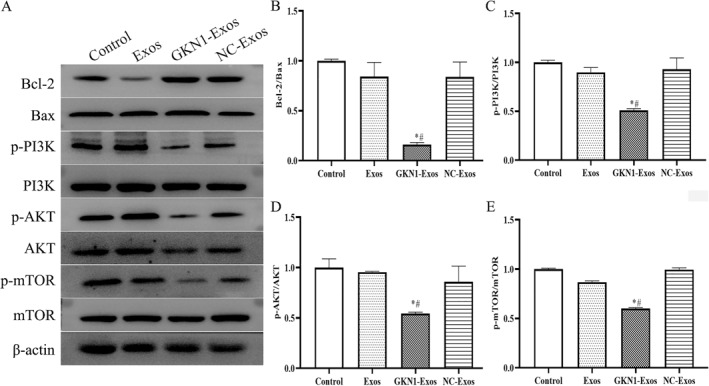
GKN1 overexpression attenuated Bcl‐2/Bax and PI3K/AKT/mTOR relative protein levels. (A) Western blot of Bcl‐2/Bax and PI3K/AKT/mTOR relative indicators in the tissue of tumor were performed; (B) Bcl‐2/Bax; (C) p‐PI3K/PI3K expression; (D) p‐ AKT/AKT expression. (E) p‐mTOR/mTOR expression. *p < 0.05 versus Control group; #*p* < 0.05 versus Exos group.

The protein levels of key components in the PI3K/Akt/mTOR signaling pathway were assessed to elucidate the potential mechanism by which GKN1 overexpression affects cell apoptosis. Analysis of Figure 8 revealed that levels of Bcl‐2/Bax, p‐PI3K/PI3K, p‐Akt/Akt, and p‐mTOR/mTOR were significantly reduced in the GKN1‐Exos group compared to the control group (*p* < 0.05). Moreover, no significant differences were observed between the NC‐exo and Exos groups when compared to the control group.

## 4 DISCUSSION

Tumor‐derived exosomes serve as mediators of intercellular communication and play a role in various cancer processes, including tumor microenvironment remodeling, immune response modulation, angiogenesis, invasion, metastasis, and drug resistance. Despite extensive research on gastric cancer‐derived exosomes dating back several decades, there is a lack of publications addressing the regulation of gastric cancer tumor suppression and a need for further elucidation of the intricate mechanisms by which exosomes contribute to gastric cancer progression and invasion.

The expression of GKN1 is downregulated in gastric tumor tissues and derived cell lines, while its overexpression in gastric cancer cell lines induces apoptosis. The measurement of GKN1 is primarily utilized for early cancer diagnosis. The mechanisms underlying GKN1's role in gastric cancer cell invasion and metastasis remain incompletely elucidated. Furthermore, there is a lack of research on GKN1‐related exosomes isolated from gastric cancer cell lines.

Both in vivo and in vitro, this study explored the effects and potential mechanisms of GC‐derived GKN1 overexpressed exosomes. In our present study, we successfully constructed GKN1 overexpressed exosomes. We found that GC‐derived GKN1 overexpressed exosomes could inhibited the cell viability and invasion rate in AGS and N87‐C cells. Through regulated PI3K/Akt/mTOR signaling, GC‐derived GKN1 overexpressed exosomes increased cell apoptosis. These results have a good consistency with the previous studies.

Studies have shown that exosomes are effective biological carriers for therapeutic factors.^28^ As a small extracellular vesicle, exosome can be released by tumor cells or immune cells. Tumor‐derived exosomes (TDEs) act as the signal transducers or messengers in cancer formation and progression.^29^ A plethora of evidence indicates that TDEs participate in the tumor angiogenesis, invasion, metastasis, and drug‐resistance within the tumor microenvironment. Previous studies revealed that exosomes from gastric cancer cells could regulate neutrophil phenotype, leading to the autophagic activation and gastric cancer cell migration.^30^, ^31^, ^32^ Exosomes from gastric cancer cells were found to promote cell viability and migration, in agreement with previous studies.

GKN1 plays a critical role in maintaining gastric mucosal integrity. Previous studies have shown that GKN1 expression is reduced in gastric cancer cells and tissues. Because of the high sensitivity and specificity of GKN1, the levels of GKN1 in serum could be regarded as informative diagnostic and gastric cancer‐specific diagnostic biomarker.^33^, ^34^ Previous studies have revealed that by downregulating MMP2 expression through the NF‐κB pathway, GKN1 inhibits metastasis in GC cells, suggesting that it may be a potential therapeutic target for gastric cancer.^35^, ^36^ Additionally, HFE‐145‐derived Exosomal GKN1 protein inhibited gastric carcinogenesis.^21^ In our present study, we successfully constructed GKN1 overexpressed exosomes. We found that GC‐derived GKN1 overexpressed exosomes could inhibited the cell viability and invasion rate meanwhile in AGS and N87‐C cells. Meanwhile, GC‐derived GKN1 overexpressed exosomes increased the cell apoptosis rate.

Tumor tissue growth and distant metastasis are largely caused by cancer cell invasion.^37^ According to previous studies, MMPs play a role in invasion, migration, metastasis, and tumorigenesis. MMP‐2 and MMP‐9 levels are known to play a major role in extracellular matrix degradation in cells.^38^ Additionally, MMP‐9 levels were found to be associated with tumor aggressiveness.^39^ The proliferation index of gastric cancer cells as determined by Ki‐67 is a measure of cancer aggressiveness. It has been confirmed that high expression of Ki‐67 and MMP‐9 contributes to gastric cancer development.^40^, ^41^ After treatment with GC‐derived GKN1 overexpressed exosomes, we found a significant decrease in cell proliferation markers Ki67 and MMP‐9.

Furthermore, we detected the indicators of Bcl‐2, Bax, caspase‐3, and caspases‐9 in response to GC‐derived GKN1 overexpression. Cell apoptosis is regulated by Bcl‐2 and Bax genes. By inhibiting cytotoxicity of Bax, Bcl‐2 regulates the opening and closing of mitochondrial membrane transition pores, intracellular calcium concentrations, and cell apoptosis.^42^, ^43^ Additionally, Bax could make mitochondria more permeable and activate apoptosis‐related proteins, thereby promoting apoptosis via Bcl‐2 heterodimerization.^44^ The balance between pro‐apoptotic and anti‐apoptotic proteins is thought to determine cell death or survival by controlling apoptosis. Caspases‐3 and caspase‐9 destroy extracellular matrix proteins and skeletal proteins, respectively, during cell apoptosis. Additionally, the cell apoptosis indicators were significantly increased. The aim of the present study was to evaluate the expression of pro‐apoptotic Bax, caspase‐3, and caspase −9, and anti‐apoptotic Bcl‐2 in cancer cells in order to understand the apoptotic pathway induced by GKN1. We found that Bax and cleaved caspase‐3 expression are highly expressed by GKN1 treatment. In this experiment, compared with the control group, GKN1 downregulated the ratio of Bcl‐2/Bax and upregulated the protein level of cleaved caspase‐3 and cleaved caspase‐9.

It has been demonstrated that the PI3K/AKT/mTOR signaling pathway play an important role in cell proliferation, migration, and invasion in several cancers, including breast and gastric cancers.^45^ Previous studies have shown that the PI3K/AKT/mTOR pathway regulates multiple cellular processes and plays a key role in gastric cancer progression.^46^ Cell proliferation and metabolism are regulated by PI3K/AKT/mTOR pathways in gastric cancer.^47^ Activated PIP3 recruits and activates AKT in the cytoplasm, thus affecting transcription factors involved in cell viability and apoptosis, which in turn affects tumorigenesis.^48^ By inhibiting PI3K/AKT/mTOR signaling, oxidative DNA damage, tumor‐specific markers, and the induction of caspase‐dependent apoptosis could be countered. Recent studies suggested that PI3K/AKT signaling pathway participates in regulating gastric cancer cells proliferation and survival. AKT has a central function in inducing cell survival by suppressing multiple downstream effectors of programmed cell death, inflammation, and mitochondria‐produced ROS. The activation of p‐PI3K, p‐AKT, and p‐mTOR was observed in gastric cancer with increasing grade. Furthermore, studies revealed that activation of PI3K/AKT/mTOR pathways was associated with invasion and angiogenesis in cells, resulting in a poor outcome.^49^, ^50^ The present study demonstrated that GC‐derived GKN1 overexpressed exosomes inhibited proliferation, invasion, and PI3K/AKT/mTOR signaling. Our results confirm these conclusions.

In this study, AGS and N87‐C cells were transfected with an overexpressed GKN1 plasmid, followed by extraction of exosomes. Our findings demonstrate that exosomes isolated from gastric cancer cell lines contain multiple functional factors, including GKN1, and that upregulation of exosomal GKN1 can inhibit the migration and invasion of gastric cancer cells. To our knowledge, this is the first study to report that exosomes carrying GKN1 overexpression from gastric cancer cells can suppress proliferation and invasion through the PI3K/AKT/mTOR signaling pathway.

As a summary, our study demonstrated that cancer‐derived exosomal GKN1 inhibited proliferation, invasion, while inhibiting the PI3K/AKT/mTOR signaling pathway. As a result of our research, we obtained mechanistic insights into GKN1's role in gastric cancer progression and developed a valuable marker to help diagnose and treat gastric cancer.

## AUTHOR CONTRIBUTIONS

Lingling Tian planned the study design, the sample cohort, supervised the study, and revised the manuscript; Li Tang gathered the subjects' data, analyzed the data and made statistical interpretations; Xu Li prepared for the Figures; Liuye Huang wrote the paper. All authors have read and agreed to the published version of the manuscript.

## CONFLICT OF INTEREST STATEMENT

The authors declare no competing interests.

## ETHICS STATEMENT

Experiments were conducted in accordance with the ARRIVE guidelines (Animal Research: Reporting of In Vivo Experiments) and we obtained approval from Yantai Yuhuangding hospital for all animal procedures.

## Data Availability

The datasets used and analyzed during the current study are available from the corresponding author on reasonable request.
